# Did the COVID-19 Pandemic Affect the Management of Patients With Acute Appendicitis?

**DOI:** 10.7759/cureus.24631

**Published:** 2022-04-30

**Authors:** Deniz Tazeoglu, Ahmet Cem Esmer, Bilal Arslan, Ahmet Dag

**Affiliations:** 1 General Surgery, Faculty of Medicine Mersin University, Mersin, TUR

**Keywords:** complication, open appendectomy, laparoscopic appendectomy, appendicitis, covid-19 pandemic

## Abstract

Background

The coronavirus disease 2019 (COVID-19) pandemic has changed the lives and habits of people all over the world. In this study, it was planned to investigate the effect of the COVID-19 pandemic on the diagnosis and treatment duration of acute appendicitis (AA), morbidity and mortality.

Methods

The data of patients who were operated on with the diagnosis of AA in our clinic between March 2019 and March 2021, divided into pre-COVID and post-COVID periods, were analyzed. Patients diagnosed with AA, who had the only appendectomy perioperatively, and who had complete preoperative blood analysis and radiological imaging data were included in the study.

Results

The time from the onset of symptoms to the time of admission to the hospital was statistically significantly longer than in the post-COVID group (p=0.04). During the COVID-19 pandemic period, the use of ultrasonography was statistically significantly reduced (p<0.01); computed tomography use increased (p<0.001). Laparoscopic appendectomy as a surgical technique decreased statistically significantly during the pandemic period (p=0.02). Postoperative complications and the postoperative complication severity degrees were not statistically significant between periods (p=0.24, p=0.68). The risk for the occurrence of postoperative complications in COVID-19 positive patients was statistically higher (p=0.01) (OR: 9.38 95% CI: 1.96 - 44.88).

Conclusion

The COVID-19 pandemic had caused delays in the admission and diagnosis of patients who might need surgery due to AA. Postoperative complication frequency and complication severity classification were not affected. COVID-19 positivity was a risk factor for complex AA presenting with periappendicular abscess, gangrenous and perforated appendix.

## Introduction

Since the first unusual cases of pneumonia were identified in China in December 2019, the novel coronavirus that causes coronavirus disease 2019 (COVID-19) has spread rapidly worldwide and caused millions of deaths around the world. On March 11, 2020, COVID-19 was declared a pandemic infection by the World Health Organisation (WHO) [1]. Health systems around the world have dedicated their energy and resources to the COVID-19 pandemic. Bed capacities were increased, new intensive care unit beds were opened, and non-urgent elective surgical procedures were postponed.

After the heavy burden of the COVID-19 pandemic on health systems worldwide, the necessity of using health resources efficiently and effectively forced the re-planning of health care services. As a result, various surgical communities globally have proposed a safe approach in which the risk of virus transmission will be minimized by applying nonoperative management whenever possible, even in emergency surgery, including acute appendicitis (AA). Other recommendations included the selective use of minimally invasive surgery and ultrafiltration systems for carbon dioxide filtration and evacuation during laparoscopy [2,3].

AA is the most common surgical emergency in the USA, with an annual incidence of 9.38 per 100,000 [4]. An acute inflammatory process characterizes most cases, but approximately 16.5% have the appendix perforated, and gangrenous or overt peritonitis is termed 'complicated appendicitis' [5]. The mortality rate for uncomplicated AA is extremely low at 0.1%; however, mortality increases with delay in admission. In addition, the risk of rupture of the appendix increases significantly 36 hours after the onset of symptoms [6].

Despite the frequency of AA, physical examination findings alone can miss the diagnosis. It has been shown that the use of ultrasonography (USG) and computed tomography (CT) improves the accuracy of diagnosis of appendicitis in some cases and reduces the number of negative appendectomies, the latter reducing the rates below 10% [7].

Although management differs in rare specific cases, the mainstay of treatment for most patients is surgery with an open or laparoscopic approach. In the UK, operative intervention is recommended for AA within 48 hours of presentation. Laparoscopic appendectomy offers clear advantages over open appendectomy, including less postoperative pain, less surgical site infection, shorter hospital stay, and faster return to normal function [8,9].

We aimed to reveal whether the COVID-19 pandemic caused a delay in diagnosing acute appendicitis, a delay in treating the disease, and whether it caused an increase in morbidity and mortality in managing the disease in the postoperative follow-up period.

## Materials and methods

Patient selection

The data of patients who were operated on with the diagnosis of AA in our clinic between March 2019 and March 2021 (Mersin University, Faculty of Medicine, Department of General Surgery) were retrospectively analyzed. Patients who were diagnosed with AA, who had the only appendectomy perioperatively, and who had complete preoperative blood analysis and radiological imaging data were included in the study. Laboratory tests (complete blood count, C-reactive peptide (CRP)) and radiological imaging methods (ultrasonography and computed tomography) data were included in the preoperative evaluation.

The study was presented to the Mersin University Faculty of Medicine Clinical Research Ethics Committee, and ethics committee approval (no 2021/517) was obtained. Helsinki Declaration rules were followed to conduct this study.

Study design

The date of March 2020, when the first COVID-19 case was detected in our country and quarantine measures were announced, was determined as the cut-off point for grouping patients. Therefore, the patients included in the study were divided into two groups according to March 2020, those who were operated on before the COVID-19 pandemic (pre-COVID) and those who were operated on during the pandemic period (post-COVID) and were compared.

Data collection

Demographic data of all patients included in the study (age, gender), onset time of complaints, preoperative laboratory values, radiological imaging methods (USG or CT), surgical technique, characteristics of perioperative appendicitis, and presence of accompanying pathology (appendiceal perforation and periappendicular abscess), radiological and pathological appendix size, appendix pathology result (normal, simple, complex), histopathological examination, presence of postoperative complications and hospital stay were recorded. The Clavien-Dindo scale was used to evaluate the severity of postoperative complications [10]. "Stratified disease approach to acute appendicitis" was used for the evaluation of the appendix.

Evaluation of acute appendicitis

The classification system developed by Carr et al. was used to classify acute appendicitis [11] (Table 1). This classification system is advantageous for surgeons and patients as it allows for gradual perioperative planning, classification of clinical severity based on preoperative assessment rather than postoperative histopathology. Classification of acute appendicitis; grouped as normal, simple, and complex.

**Table 1:** Stratified disease approach to acute appendicitis Modified from the classification system by Carr [11]

Pattern	Macroscopic appearances	Microscopic appearances	Clinical relevance
Normal appendix			
Normal underlying pathology	No visible changes	Absence of any abnormality	Consider other causes
Acute intraluminal inflammation	No visible changes	Luminal neutrophils only with no mucosal abnormality	Might be the cause of symptoms. but consider other causes
Acute mucosal/submucosal inflammation	No visible changes	Mucosal or submucosal neutrophils and/or ulceration	Might be the cause of symptoms. but consider other causes
Simple. non-perforated appendicitis			
Suppurative/phlegmonous	Congestion. colour changes. increased diameter. exudate. pus	Transmural inflammation. ulceration. or thrombosis. with or without extramural pus	Likely cause of symptoms
Complex appendicitis			
Gangrenous	Friable appendix with purple. green. or black colour changes	Transmural inflammation with necrosis	Impending perforation
Perforated	Visible perforation	Perforation; not always visible in microscope	Increased risk of postoperative complications
Abscess (pelvic/abdominal)	Mass found during examination or abscess seen on preoperative imaging; or abscess found at surgery	Transmural inflammation with pus with or without perforation	Increased risk of postoperative complications

Statistical analysis

Parametric tests were used without the normality test due to the compatibility of the Central Limit Theorem. In the analysis of the data, the mean and standard deviation, minimum and maximum values of the features; frequency and percentage values were used to define categorical variables. The student's t-test statistic was used to compare the means of two independent groups, and the one-way ANOVA test statistic was used to compare the means of more than two groups. If a difference was detected with ANOVA, it was evaluated with Tukey statistics as a post-hoc test. Chi-square test statistics were used to evaluate the relationship between categorical variables. The statistical significance level of the data was taken as p<0.05. In the evaluation of the data, www.e-picos.com New York software and MedCalc statistical package program were used.

## Results

During the study, 229 of 253 patients diagnosed with acute appendicitis, who met the inclusion criteria, were included. Eleven patients with incomplete results of preoperative blood tests and radiological imaging methods, two patients who underwent segmental ileum resection for Meckel's diverticulitis with appendectomy, four patients who underwent percutaneous drainage due to plastron appendicitis + periappendicular abscess, and seven patients who refused to be operated and were treated with oral antibiotics were excluded. Of the patients included in the study, 132 (57.6%) were divided into two groups as those operated before the pandemic (pre-COVID) and 97 (42.4%) during the pandemic period (post-COVID) (Table 2).

**Table 2:** The effect of the pandemic process on the socio-demographic and clinical status of the patient WBC: white blood cell. CRP: C-reactive peptide. USG: Ultrasonography. CT: Computed tomography

	All patient (n=229)	Pre-COVID (n=132)	Post-COVID (n=97)	p value
	x̄±SD	x̄±SD	x̄±SD	
Age	37.5±13	36.9±13.1	38.4±13	0.4
WBC	13.21±3.21	13.38±3.28	12.97±3.11	0.33
Neutrophil	10.14±2.46	10.25±2.5	9.99±2.42	0.43
CRP	67.44±41.38	67.33±42.8	67.54±39.6	0.96
Appendix size	9.92±1.73	9.81±1.69	10.07±1.71	0.25
	n (%)	n (%)	n (%)	
Gender				
Female	100 (43.7)	58 (43.9)	42 (43.3)	0.92
Male	129 (56.3)	74 (56.1)	55 (56.7)	
Admission time				
1. day	164 (71.6)	102 (77.3)	62 (63.9 )	0.04
2. day	47 (20.5)	24 (18.2)	23 (23.7)	
3. day	18 (7.9)	6 (4.5)	12 (12.4)	
Abdominal USG				
Yes	136 (59.4)	88 (66.7)	48 (49.5)	0.009
No	93 (40.6)	44 (33.3)	49 (50.5)	
Abdominal CT				
Yes	112 (48.9)	44 (33.3)	68 (70.1)	<0.001
No	117 (51.1)	88 (66.7)	29 (29.9)	
Preoperative Covid-19 Test				
Yes	-	-	91 (93.8)	-
No	-	-	6 (6.2)	
Covid-19 test result				
Positive	-	-	12 (13.2)	-
Negative	-	-	79 (86.8)	

Preoperative nasopharyngeal swab samples were taken from 91 (93.8%) patients operated on during the pandemic period, and the COVID-19 PCR test was performed. While the COVID-19 test was negative in 79 (86.8%) patients, it was positive in 12 (13.2%) patients.

The duration from the onset of AA-related complaints to the patients’ admission to the hospital was between one to three days in both groups. On the first day (early), the rate of hospital admissions was 77.3% in the pre-COVID group, compared to 63.9% in the post-COVID group. On the third day (late), the rate of hospital admissions was 4.5% in the pre-COVID group, compared to 12.4% in the post-COVID group. Comparison between early and late admission revealed that admission time to the hospital was statistically significantly longer in the post-COVID group (p=0.04).

When evaluated according to laboratory values, no statistically significant difference was found between leukocyte, neutrophil, and CRP levels in the pre-COVID and post-COVID groups.

When evaluated according to the use of radiological imaging methods, USG was performed in 67% (n=88) of the patients in the pre-COVID group and 49% (n=48) in the post-COVID group. CT scan was performed in 33% (n=44) of patients in the pre-COVID group and 70% (n=68) in the post-COVID group. For the radiological evaluation of the width and features of the appendix, during the COVID-19 pandemic period, the use of USG was statistically significantly reduced; CT use increased (p<0.001).

When the appendix was evaluated radiologically, the thickness of the appendix was 9.81±1.69 cm in the pre-COVID group; it was 10.07±1.71 cm in the post-COVID group. There was no significant difference between the groups in terms of appendix width (p=0.25) (Table 2).

Laparoscopic appendectomy was performed in 55.3% of patients in the pre-COVID group and 40.2% of patients in the post-COVID group. It was observed that the preference for laparoscopic appendectomy as a surgical technique decreased during the pandemic period (p=0.02) (Table 3).

**Table 3:** Comparison of the postoperative conditions and results of the patients according to the pandemic process

	All patient (n=229)	Pre-COVID (n=132)	Post-COVID (n=97)	p value
	n (%)	n (%)	n (%)	
Surgical Technique				
Laparotomy	117 (51.1)	59 (44.7)	58 (59.8)	0.02
Laparoscopy	112 (48.9)	73 (55.3)	39 (40.2)	
Perioperative evaluation of acute appendicitis				
Normal	13 (5.6)	9 (6.8)	4 (4.2)	0.08
Simple	168 (73.4)	102 (77.3)	66 (68)	
Complex	48 (21)	21 (15.9)	27 (27.8)	
Perforated appendicitis				
Yes	36 (15.7)	20 (15.2)	16 (16.5)	0.78
No	193 (84.3)	112 (84.8)	81 (83.5)	
Periappendicular abscess				
Yes	12 (5.2)	5 (3.8)	7 (7.2)	0.25
No	217 (94.8)	127 (96.2)	90 (92.8)	
Postoperative complication				
Yes	16 (6.9)	7 (5.3)	9 (9.3)	0.24
No	213 (93.1)	125 (94.7)	88 (90.7)	
Clavien Dindo				
Grade 1	10 (62.5)	4 (57.1)	6 (66.7)	0.68
Grade 2	2 (12.5)	1 (14.3)	1 (11.1)	
Grade 3	3 (18.8)	1(14.3)	2 (22.2)	
Grade 4	-	-	-	
Grade 5	1 (6.3)	1(14.3)	-	
Hospital stay time				
1 day	172 (75.1)	103 (78)	69 (71.1)	0.23
≥2 day	57 (24.9)	29 (22)	28 (28.9)	
Negative appendectomy	23 (10)	18 (13.6)	5 (5.2)	0.04

According to the preoperative evaluation, 73.4% of the patients were in the simple class, 21% in the complex class, and 5.6% in the normal class. There was no significant difference between the pre-COVID and post-COVID groups according to the classification of the appendix (p=0.08). During appendectomy, gangrenous appendicitis was detected in 5.2% (n=12), perforation appendicitis in 15.7% (n=36), and periappendicular abscess in 5.2% (n=12). There was no significant difference between the groups regarding the incidence of gangrenous appendicitis, perforated appendicitis, and periappendicular abscess (Table 3).

When postoperative complications are evaluated, postoperative complications occurred in seven (5.3%) patients in the pre-COVID group and nine (9.3%) patients in the post-COVID group. Wound infection was detected in 10 patients, the intra-abdominal abscess was detected in five patients, and one patient passed away postoperatively. Antibiotherapy was sufficient in two patients to treat patients with intra-abdominal abscesses; a percutaneous drainage catheter was inserted in three patients. There was no statistically significant difference between the groups in terms of postoperative complications (p=0.24). There was no significant difference between the groups according to the classification of postoperative complication severity (p=0.68) (Table 3).

When the presence of COVID-19 disease and the association of postoperative complications were evaluated, complications were seen in 33% of COVID-19 positive patients, while it was seen in 5.1% of negative patients. Thus, the risk for the occurrence of postoperative complications in COVID-19 positive patients was statistically higher than the risk in COVID-19 negative patients (p=0.01; OR: 9.38 95% CI: 1.96 - 44.88) (Table 4).

**Table 4:** Risk factors affecting the occurrence of postoperative complications

	Odds Ratio	%95 CI	p value
Gender	0.99	0.36 – 2.78	>0.05
Covid-19 outbreak	1.83	0.66 – 5.09	>0.05
Covid positivity	9.38	1.96 – 44.88	0.01
Perforated appendicitis	16.54	5.31 – 51.55	<0.001
Periappendicular abscess	32.36	8.58 – 122.04	<0.001

In the occurrence of postoperative complications, while COVID-19 positivity, presence of periappendicular abscess, gangrenous and perforated appendix were risk factors, gender was not. Although performing appendectomy during the pandemic increased the risk of complications, there was no statistical difference (Table 4).

The hospital stay after appendectomy was one day in 172 (75.1%) of the patients; two or more in 57 (24.9%) of them. There was no significant difference between the groups regarding the length of hospital stay (p=0.23) (Table 3).

Histopathological examination was performed on all patients after appendectomy. A negative appendectomy was performed in 18 (13.6%) patients in the pre-COVID group and in five (5.2%) patients in the post-COVID group. The number of negative appendectomy patients was statistically significantly decreased in the post-COVID group compared to the pre-COVID group (p=0.04) (Table 3). The risk of detecting a negative appendectomy in patients who underwent surgery in the post-COVID period was statistically lower than the risk in patients who underwent surgery in the pre-COVID period (p<0.05; OR: 0.34 95% CI: 0.12 - 0.96).

## Discussion

COVID-19 is a severe health problem that has affected millions worldwide since December 2019. At the beginning of the pandemic, the lack of sufficient information and evidence about the transmission, treatment, and prevention of the disease and the negative images reflected in the media created fear and panic among the people [12]. As a result, many patients began to avoid hospitals as much as possible for fear of contracting the COVID-19 virus.

There were delays in the admission and diagnosis of medical conditions such as appendicitis, which we frequently encounter in daily clinical practice and require urgent surgical treatment. Although the main symptoms of COVID-19 are respiratory problems, gastrointestinal symptoms such as abdominal pain, diarrhea, nausea, and vomiting that may be confused with acute appendicitis symptoms have also been reported [13]. AA continues to be one of the most important causes of acute abdominal pain in adults worldwide and is one of the most common reasons for emergency surgery [14].

The mortality rate from uncomplicated AA is extremely low at 0.1%; however, mortality increases with delay in admission [15]. The risk of rupture of the appendix increases significantly 36 hours after the onset of symptoms. Gangrenous appendicitis occurs in 10% of patients, and perforation or abscess occurs in up to one-fifth, both of which are associated with increased postoperative complications [16].

In our study, the meantime from the onset of complaints to the time of admission to the hospital was found to be one to three days, with pre-COVID and post-COVID periods. However, in the post-COVID period, the number of patients admitted to the hospital on the first day decreased significantly compared to the pre-COVID period, and the rate of patients admitted on the third day of the complaints increased significantly. We think this results from patients avoiding the hospital for fear of contracting COVID-19 at the beginning of their complaints and applying to the hospital when their complaints become increasingly unbearable. However, this situation can inevitably cause the disease to become dangerous and mortal when patients wait until their complaints become increasingly unbearable.

Physical examination findings, laboratory tests, and imaging methods are used to diagnose AA. As the primary imaging method, USG is generally sufficient and widely used. However, CT is used for definitive diagnosis when USG is insufficient and other pathologies are suspected. Therefore, it can be said that there are changes in the order and frequency of use of diagnostic tools in order to diagnose AA all over the world during the COVID-19 pandemic process. Although there are not many studies evaluating this situation, in a study by Romero et al. in which AA patients were evaluated during the COVID-19 period, it was reported that the CT scan method applied to these patients decreased by 57%, and the severity of the disease was found to be increased [17]. In our study, while the use of USG decreased statistically significantly after post-COVID, CT use increased.

We think that this situation is caused by the desire of surgeons to make a definitive diagnosis with higher sensitivity and simultaneously evaluate their lungs for COVID-19 involvement in AA patients. However, while the extraordinary nature of the pandemic period makes this approach feasible, it is clear that it is not feasible under normal conditions due to its non-cost-effectiveness and high radiation exposure.

Although the nonoperative approach in AA treatment has been discussed recently, it has been reported that it can only be applied in uncomplicated appropriate patients. Treatment of AA is open or laparoscopic appendectomy. Laparoscopic appendectomy is the gold standard treatment option for less pain and shorter hospital stay [16]. However, although there is no evidence that COVID-19 can be spread by pneumoperitoneum and aerosolization via smoke during minimally invasive surgery, this risk cannot be excluded [18].

In a study conducted by Ielpo et al., in which 709 surgeons participated and the attitudes of surgeons in Europe towards AA patients during the post-COVID period were evaluated, it was revealed that one-third of the surgeons preferred open appendectomy, considering the high risk of contamination, without evidence-based recommendations by the surgical community. [18]. In the guideline published by the American College of Surgeons, it was suggested that surgeons should avoid laparoscopic surgery for optimum protection from COVID-19 transmission [19]. In our study, we observed that the preference for laparoscopic appendectomy as a surgical technique decreased statistically significantly in the post-COVID period (p=0.02). We think that the risk of transmission may be high due to airborne contamination of the disease, hanging in the air, and insufflation of the abdomen with CO2 in laparoscopy, which are the factors that cause surgeons to prefer open surgery.

AA can evolve rapidly with 16% to 40% perforation rates and more frequent complications in younger age groups and patients over 50. Appendicular perforation is associated with increased morbidity and mortality compared with acute nonperforated appendicitis. Mortality rates in perforated appendicitis are 5% [20]. Temple et al., in a prospective study, showed that late admission to hospital was associated with higher perforation rates. Accordingly, the longer the patients wait to go to the hospital, the higher the probability of perforation [21]. Moreover, following this finding, a higher proportion of patients who underwent an appendectomy at post-COVID-19 period presented complicated appendicitis (8.0% vs. 42.5%, p<0.001) in a study by Cano-Valderrama et al. [22]. In the definition made by Carr, the definition of complex appendicitis was evaluated as appendicitis becoming gangrenous, perforated, or abscess formation (pelvic or abdominal) after the inflammation process [11].

In our study, inconsistent with these studies, there was no significant difference in the incidence of gangrenous appendicitis, perforated appendicitis, and periappendicular abscess between the pre-COVID and post-COVID periods. Again, in our study, when postoperative complications were compared and the postoperative complication severity classification was evaluated according to the Clavien-Dindo scale, no statistically significant difference was observed in the pre-COVID and post-COVID periods (p=0.24, p=0.68). This result favors the fact that there was no difference in complication rates, although there was a delay in the admission and diagnosis of the patients during the pandemic period. Another result that supports this conclusion is that there is no statistically significant difference between the two periods in the length of hospital stay of the patients (p=0.23). The opposite opinion is that non-complex disease (normal or simple appendicitis) heals in many patients without surgical intervention by limiting itself during the curfews during the pandemic process [23]. However, in our study, positivity for COVID-19 was a risk factor for complex AA presenting with periappendicular abscess, gangrenous and perforated appendix. Therefore, we think that COVID-19 positivity aggravates the course of the disease, albeit partially. This situation may be caused by hospital avoidance behavior or inflammatory processes. Although it is not the subject of this study, it has been suggested that the treatment of non-complex, normal, and simple AA can be done with the non-surgical method. Many studies supporting this issue have been published during the pandemic process [24]. The results of our study show that despite the delay in admissions to the hospital, there is no significant difference in the complication rates of the disease in terms of the period and severity.

It is thought that the rate of negative appendectomy decreases only the patients with severe symptoms admitted to hospital during the pandemic period, cross-sectional radiological imaging methods were used more frequently, and the surgeons were more meticulous in appropriate patient selection for surgery. However, despite all this, it should not be ignored that there may be acute appendicitis that may be overlooked due to the fear caused by the COVID-19 pandemic.

In extraordinary times of chaos and turmoil, alternatives to evidence-based treatment methods may necessarily emerge. These early findings suggest that established treatment habits for the management of AA during and perhaps beyond the COVID-19 pandemic may evolve in a new direction. Non-surgical options may be considered to manage uncomplicated AA soon. However, more evidence-based studies are needed on this subject.

## Conclusions

The COVID-19 pandemic has caused a delay in the admission and diagnosis of patients who may need surgery due to AA, the use of CT in the diagnosis process has increased, and the open surgical approach has been preferred more often. The COVID pandemic did not affect the incidence of postoperative complications after AA. COVID-19 positivity can be a risk factor for complex AA presenting with periappendicular abscess, gangrenous and perforated appendix.
